# Comparison of Swedish nurses’ attitudes toward involving families in care over a decade

**DOI:** 10.1186/s12912-022-00827-z

**Published:** 2022-03-02

**Authors:** Hanne Konradsen, Zarina Nahar Kabir, Anne-Marie Boström, Kristofer Årestedt

**Affiliations:** 1 Department of Gastroenterology, Herlev and Gentofte Hospital, Borgmester Ib Juuls Vej 1, 2730 Herlev, Denmark; 2 Department of Clinical Medicine, Faculty of Health and Medical Sciences, University of Copenhagen, Copenhagen, Denmark; 3 Department of Neurobiology, Care Sciences and Society, Division of Nursing, Karolinska Institute, Stockholm, Sweden; 4 Theme Inflammation and Aging, Karolinska University Hospital, Huddinge, Sweden; 5 R&D Unit, Stockholms Sjukhem, Stockholm, Sweden; 6 Faculty of Health and Life Sciences, Linnaeus University, Växjö, Sweden; 7 The Research Section, Region Kalmar County, Kalmar, Sweden

**Keywords:** Attitudes, Care resources, Family, Nursing, Sweden

## Abstract

**Background:**

Involving families in care benefits both patients and their families. Sweden was one of the first countries to introduce family nursing, but its effect on nurses’ attitudes toward involving families in care was unknown. First, this study aimed to investigate registered nurses’ attitudes about the importance of involving families in nursing care. Second, it aimed to compare these attitudes over a decade.

**Methods:**

This comparative study was based on data from two separate studies. Data were collected using the Families Importance in Care – Nurses’ Attitudes questionnaire. The first phase of data collection took place in 2009, and the second phase was conducted in 2019.

**Results:**

Overall, the nurses were positive towards involving families in care, both in 2009 and 2019. Overall, no significant difference was found between the two studies from 2009 and 2019. On a subscale level, nurses reported significantly higher levels on *family as a resource* in the study from 2009 compared to the study from 2019. The opposite was shown for the subscales *family as a burden* and *family as an own resource*. According to the *R*^2^ values (0.002 – 0.04), the effect sizes were small.

**Conclusion:**

In Sweden, nurses’ attitudes toward involving families in care did not change over the studied decade, despite changes in nursing, healthcare-system, and society.

## Introduction

Growing evidence has suggested that patients’ families play an important role during the illness process and that family interactions and strengthened relations benefit both patients and their families [1, 2]. Family members might take on various supporting roles, such as accompanying patients to healthcare appointments and procedures, providing emotional support, providing practical tasks, and supporting patients’ decision-making [3].

### Background

Earlier studies have found that nurses consider families both as a resource and, sometimes, as a challenge [4, 5]. When families are considered a resource, nurses find family involvement to be part of their job and important in delivering good care [5]. Less supportive attitudes toward families in nursing care have related to a difference in culture, demanding families, or suffering families [6, 7]. A recent integrative review found that nurses’ attitudes help or hinder families’ involvement in care [8]. Family involvement in care and decision-making can be complex. Healthcare professionals might lack education in how to involve families, and they might also lack sufficient time to involve families in care [3, 9]. Nurses’ attitudes toward involving families may also influence the level of family involvement that occurs, and less positive attitudes toward this involvement may make families feel excluded, decreasing their confidence about participating in care [8, 9].

Nursing education in Sweden was raised to the university level in 1977, and in 1992, it became a three-year program at the bachelor level [10]. A 2020 study found that Swedish nursing students harbor positive expectations about nurses’ role and expect teamwork, as well as possibilities to develop professional and continued work satisfaction [11]. Sweden has been one of the first countries to introduce family nursing. In 2010, Saveman published a synthesis of Swedish family nursing research, locating 75 family nursing studies that had originated in Sweden [12]. Saveman concluded that, despite this strong foundation, only a few studies had examined interventions, and the work of implementing family nursing into practice was still needed, including collaboration across research, clinical practice, and education.

The “Families’ Importance in Nursing Care – Nurses’ Attitudes” (FINC-NA) instrument was developed in Sweden, based on a literature review [13]. The questionnaire was used to collect data about Swedish registered nurses (RNs) who had been randomly selected from a registry of members of the Swedish Association of Health Professional Nurses [14]. The study found that nurses generally had positive attitudes toward families’ involvement—but also that newly graduated nurses had found family cooperation difficult. A recent review investigated instruments assessing nurses’ attitudes toward involving families in clinical practice [15]. Five instruments were identified, and based on the COSMIN checklist, the FINC-NA scored highly in its psychometric properties; it was the only identified instrument that had incorporated a generic nursing perspective. The questionnaire has since been translated into several different languages and tested among nurses working in Denmark [16], Belgium [17], Portugal, Taiwan [18], Australia [8], Brazil [19], Iceland [20], and other countries.

During the last decade, several major changes have been implemented in Swedish healthcare. For example, the diagnosis and treatment of various diseases have been transferred from hospital care to outpatient clinics or primary care. The number of hospital beds in the country decreased from 31,765 in 2000 to 21,754 in 2018 [21]. The country’s average length of hospital stays decreased from 7.1 days in 2010 to 5.7 days in 2017 [22]. The number of beds in nursing homes or residential care settings decreased even as the number of older people increased. Older people with complex needs and health problems are, to a greater extent, living at home with support from home healthcare and home care services [23]. These changes imply that patients are expected to care for themselves at home with support from family members or relatives, which means nurses must prepare not only patients but also family members or relatives for at-home patient support. To support and to secure the health and wellbeing of both older patients and their family, nurses have to work more pro-active family-focused [24].

Therefore, examining whether nurses’ attitudes toward involving family members in nursing care have changed to become more positive is worthwhile.

## The study

### Aim

First, the current study aimed to investigate RNs’ attitudes about the importance of involving families in nursing care. Second, it aimed to compare these attitudes over a decade.

### Design

This comparative study was based on data from two separate studies conducted ten years apart. The first phase of data collection took place in 2009 and was reported in a study by Saveman et al. (2011). The second phase of data collection was conducted for the present study 2019.

#### The 2009 study

In 2009, data were collected by randomly distributing a questionnaire to members of the Swedish Association of Health Professional Nurses who were also studying at an advanced level at four different universities in Sweden. The questionnaire included the FINC-NA and background questions, and it was sent by mail to 746 nurses. In total, 246 nurses completed the questionnaire. More details about the study can be found in the study by Saveman et al. (2011).

#### The 2019 study

##### Sample/participants

The current study’s participants were registered nurses living in Sweden. A snowball sampling technique was used through Facebook and other social media platforms. An invitation to complete the questionnaire was sent through personal networks, identified Facebook groups, and professional Facebook groups related to nursing. The invitation also included a request for respondents to only complete the survey once and to send the questionnaire to other nurses they knew through Facebook contacts or other electronic contacts.

##### Data collection

All participants were asked about their age, gender, and personal experience with a seriously ill family member in their own families. Next, they were asked to complete a web-based version of the FINC-NA. This online version did not allow respondents to progress to the survey’s next question before answering its preceding question, ensuring that no data were missing.

The final version of the FINC-NA questionnaire comprises 26 items, which were answered on a five-point Likert-type scale with anchored endpoints (from 1 = *strongly disagree* to 5 = *strongly agree*). The instrument was originally developed as a unidimensional scale, but it has been shown to be a multidimensional scale [13, 25]. The instrument includes four subscales: *familiy as a resource in nursing care* (Fam-RNC; 10 items exploring the perception of family presence during care and family participation in care; a score ranging from 10 to 50), *family as a conversational partner* (Fam-CP; eight items exploring the perception of communication and dialog between families and nurses; a score ranging from 8 to 40), *family as a burden* (Fam-B; four items exploring negative attitudes toward involving families in care; a score ranging from 4 to20), and *family as its own resource* (Fam-OR; four items exploring perceptions of families using their own resources and opportunity to cope with an illness situation; a score ranging from 4 to 20). The scores in the Fam-B subscale were reversed before analysis so that all the subscales were comparable. A respondent’s total score was the sum of all response items, with a possible range between 26 and 130. For all scales, a higher score indicated a more positive attitude.

##### Ethical considerations

Ethical permission to conduct this study was obtained in 2019 from the Swedish Regional Ethical Review Authority (number 2018/1535–31). Facebook and other social media do not necessarily present a clear boundary between *private* and *public* information. This study, therefore, did not collect data from Facebook profiles but, rather, only data provided by participants themselves by completion of the questionnaire. We were unable to identify which participants had sent the questionnaire to other prospective participants and which had not.

### Data analysis

Descriptive statistics were used to present the characteristics of the two samples and RNs’ attitudes about the importance of including families in nursing care. The two samples were compared using an independent sample *t*-test and a chi-square test.

To examine within-group differences between the various FINC-NA scales, a repeated one-way ANOVA (analysis of variance) was conducted. To make the subscale scores comparable, they were first linearly transformed into a possible score between 1 and 5. Their effect size was assessed with a partial eta squared (η^2^) approach. Cohen (1988) suggested the following interpretation: 0.02 as *small*, 0.13 as *medium*, and 0.26 as *large*. Dependent-sample *t*-tests were used as a post hoc analysis. To decrease the risk of type-I errors, these post hoc analyses used Bonferroni corrected *p*-values (*p* < 0.008).

Differences in attitudes about the importance of including families in nursing care between the two samples were examined using nested linear regression analyses, with one regression model for each FINC-NA scale (the outcome variables). These regression analyses were conducted in two steps. In step I, the sample (0 = *2019*, 1 = *2009*) was included as a single explanatory variable. In step II, age (continuous), sex (0 = *male*, 1 = *female*) and personal experience with a seriously ill family member in a participant’s own family (0 = *no*, 1 = *yes*) were entered as adjusting covariates. The effect size was assessed with Cohen’s *f*^2^ using the following interpretation: 0.02 as *small*, 0.15 as *medium*, and 0.35 as *large* [26].

The significant level was set at *p* < 0.05, and statistical analyses were conducted in Stata 16.1 (StataCorp LLC, College Station, TX, USA).

### Validity and reliability/Rigor

A recent review found that sampling through Facebook is well suited to collecting data across cultural differences, and other researchers have found that it enables a higher number of potential participants over a shorter time than conventional data collection methods [27].

The FINC-NA has demonstrated satisfactory measurement properties [25]. The present study’s internal consistency was satisfactory, according to its Cronbach’s alpha values, which ranged between 0.73 and 0.92 for the 2009 sample and between 0.75 and 0.92 for the 2019 sample.

## Results/findings

### Characteristics of the two registered nurse samples

Characteristics of the two samples of RNs are presented in Table 1. The 2009 and 2019 samples included 246 and 363 RNs, respectively. Most participants in both samples were women (92% vs. 93%, *p* = 0.478). The 2009 sample’s mean age was significantly lower than the 2019 sample’s (36.2 vs. 42.2, *p* < 0.001). In the 2009 sample, significantly fewer RNs reported having had personal experiences with a seriously ill member of their own families (66% vs. 83%, *p* < 0.001).

**Table 1:** Characteristics of the two cohorts of registered nurses from 2009 and 2019 (*n* = 609)

	2009 (*n* = 246)	2019 (*n* = 363)	*p*-value
Age (years), mean (SD)	36.2 (8.2)	42.2 (10.0)	< 0.001 ^a^
Gender, *n* (%)			0.478 ^b^
Female	226 (91.9)	339 (93.4)	
Male	20 (8.1)	24 (6.6)	
Personal experience with a seriously ill own-family member, *n* (%)			< 0.001 ^b^
Yes	161 (65.5)	301 (82.9)	
No	85 (34.6)	62 (17.1)	

^a^ Independent sample *t*-test

^b^ Chi-square test

### Descriptions of the registered nurses’ attitudes

The RNs’ attitudes about the importance of including families in nursing care and within-group comparisons are presented in Table 2. In the pooled sample, the highest levels of positive attitudes were reported in the Fam-CP subscale, followed by Fam-RNC, Fam-B, and Fam-OR (*p* < 0.001). The post hoc test showed significant differences between all subscales except Fam-RNC versus Fam-CP.

**Table 2:** Registered nurses’ attitudes about the importance of including families in nursing care and within-group comparisons (FINC-NA scale scores transformed into a common 1–5 scale)

Cohort	Fam-RNC, Mean (SD)	Fam-CP, Mean (SD)	Fam-B, Mean (SD)	Fam-OR, Mean (SD)	*p*-value^a^	ES (*η*^2^)^b^	Post hoc test^c^
All (*n* = 609)	4.05 (0.56)	4.07 (0.67)	3.95 (0.83)	3.83 (0.79)	< 0.001	0.05	-BCDEF
2009 (*n* = 246)	4.19 (0.56)	3.97 (0.68)	3.78 (0.79)	3.67 (0.79)	< 0.001	0.18	ABCDE-
2019 (*n* = 363)	3.95 (0.55)	4.14 (0.66)	4.07 (0.83)	3.94 (0.77)	< 0.001	0.04	AB–EF

^a^ Repeated one-way ANOVA (analysis of variance)

^b^ Partial eta squared effect size: 0.02 = *small*, 0.13 = *medium*, 0.26 = *large*

^c^ Repeated dependent sample *t*-test with Bonferroni corrected *p*-values (*p* < 0.008): A = Fam-RNC ≠ Fam-CP, B = Fam-RNC ≠ Fam-B, C = Fam-RNC ≠ Fam-OR, D = Fam-CP ≠ Fam-B, E = Fam-CP ≠ Fam-OR, F = Fam-B ≠ Fam-OR

In the 2009 sample, the highest levels of positive attitudes were reported in the Fam-RNC subscale, followed by Fam-CP, Fam-B, and Fam-OR (*p* < 0.001). The post hoc test showed significant differences between all subscales except for Fam-RNC versus Fam-CP.

In the 2019 sample, the highest levels of positive attitudes were reported in the Fam-CP subscale, followed by Fam-B, Fam-RNC, and Fam-OR (*p* < 0.001). The post hoc test showed significant differences between all subscales except for Fam-RNC versus Fam-OR and Fam-CP versus Fam-B.

The effect size was small for the polled sample (*η*2 = 0.05) and 2019 sample (*η*2 = 0.04). Meanwhile, the effect size was moderate for the 2009 sample (*η*2 = 0.18).

### Differences in registered nurses’ attitudes between the two samples

Differences in RNs’ attitudes about the importance of including families in nursing care between the two samples are presented in Table 3. The unadjusted regression models (Step I) showed that the 2009 sample reported significantly higher levels of positive attitudes about the importance of including family members in nursing care regarding the Fam-RNC subscale (B = 2.36, *p* < 0.001). This difference remained in the adjusted model (Step II)—that is, when *age*, *sex*, and *own experience of a seriously ill family member* were added to the model (B = 3.06, *p* < 0.001). In contrast, the 2009 sample reported significantly lower levels of positive attitudes regarding the Fam-CP (B = -1.36, *p* = 0.002), Fam-B (B = -1.17, *p* < 0.001) and Fam-OR (B = -1.08, *p* < 0.001) subscales in the unadjusted models. In the adjusted models, these differences remained for the Fam-B (B = -0.66, *p* = 0.018) and Fam-OR (B = -0.64, *p* = 0.016) subscales but not for the Fam-CP (B = -0.37, *p* = 0.404) subscale. No significant difference was found for the FINC-NA total scale before or after the adjusting covariates were added to the regression model (B = -1.25, *p* = 0.301 vs. B = 1.39, *p* = 0.260). For the significant and unadjusted regression models, the *R*^2^ values ranged between 0.02 and 0.04 which reflects a small effect size.

**Table 3:** Linear regression models to detect differences in attitudes about the importance of including families in nursing care between the 2011 and 2019 cohorts (*n* = 609)

		Step I: Unadjusted models		Step II: Adjusted models	
Outcome variable	Explanatory variable	B (se)^a^	*p*-value	B (se)	*p*-value
Fam-RNC	2009	2.36 (0.46)	< 0.001	3.06 (0.47)	< 0.001
	Age			0.10 (0.02)	< 0.001
	Female			2.59 (0.85)	0.002
	Personal experience			0.45 (0.53)	0.397
	Model statistics:	F(1, 607) = 26.78, *p* < 0.001, *R*^2^ = 0.04		F(4, 604) = 14.65, *p* < 0.001, *R*^2^ = 0.09	
	ES (*f*^2^):^b^	0.04		0.10	
Fam-CP	2009	-1.36 (0.44)	0.002	-0.37 (0.44)	0.404
	Age			0.14 (0.02)	< 0.001
	Female			3.47 (0.80)	< 0.001
	Personal experience			0.60 (0.49)	0.221
	Model statistics:	F(1, 607) = 9.53, *p* = 0.002, *R*^2^ = 0.02		F(4, 604) = 19.72, *p* < 0.001, *R*^2^ = 0.12	
	ES (*f*^2^):^b^	0.02		0.14	
Fam-B	2009	-1.17 (0.27)	< 0.001	-0.66 (0.28)	0.018
	Age			0.08 (0.01)	< 0.001
	Female			0.20 (0.50)	0.690
	Personal experience			0.01 (0.31)	0.979
	Model statistics:	F(1, 607) = 18.88, *p* < 0.001, *R*^2^ = 0.03		F(4, 604) = 14.55, *p* < 0.001, *R*^2^ = 0.09	
	ES (*f*^2^):^b^	0.03		0.10	
Fam-OR	2009	-1.08 (0.26)	< 0.001	-0.64 (0.27)	0.016
	Age			0.06 (0.01)	< 0.001
	Female			0.48 (0.48)	0.319
	Personal experience			0.31 (0.30)	0.290
	Model statistics:	F(1, 607) = 17.88, *p* < 0.001, *R*^2^ = 0.03		F(4, 604) = 11.53, *p* < 0.001, *R*^2^ = 0.07	
	ES (*f*^2^):^b^	0.03		0.08	
Fam-Total	2009	-1.25 (1.21)	0.301	1.39 (1.23)	0.260
	Age			0.38 (0.06)	< 0.001
	Female			6.73 (2.21)	0.002
	Personal experience			1.38 (1.37)	0.316
	Model statistics:	F(1, 607) = 1.07, *p* = 0.301, *R*^2^ < 0.01		F(4, 604) = 14.20, *p* < 0.001, *R*^2^ = 0.09	
	ES (*f*^2^):^b^	< 0.01		0.10	

*FINC-NA* “Families’ Importance in Nursing Care-Nurses’ Attitudes”, *Fam-RNC* family as a resource in nursing care, *Fam-CP* family as a conversational partner, *Fam-B* family as a burden, *Fam-OR* family as its own resource

^a^ Unstandardized slope coefficient

^b^ Cohens *f*^2^ effect size: 0.02 = *small*, 0.15 = *medium*, 0.35 = *large*

Among the adjusting covariates, higher ages were significantly associated with higher levels of positive attitudes in all FINC-NA subscales. In contrast, personal experience with a seriously ill family member in a respondent’s own family was not associated with any of the FINC-NA subscales. Female gender was associated with higher levels of positive attitudes in the Fam-RNC and Fam-CP subscales, as well as the FINC-NA total scale, but not in the Fam-B or Fam-OR subscales.

## Discussion

In this study, we compared data on Swedish nurses’ attitudes toward involving families in care over a period of ten years. Overall, nurses were found to have held a positive attitude toward families’ importance in care at both data collection years, and no significant difference was found in the FINC-NA total scale between the two measurement times. Such general positive attitudes have also been found in studies conducted in other countries around Europe, such as the Netherlands and Denmark [16, 17].

Little had changed in nurses’ attitudes toward involving families in care from 2009 to 2019 in Sweden, and only small changes were detected. Among the 2019 participants, fewer positive attitudes were found in relation to including family members in care, whereas more positive attitudes were found in relation to perceiving families as conversational partners, burdens, and its own resource compared with the participants who had responded ten years previously. Further research is needed in order to fully understand these complex changes, preferably by undertaking explorative studies of qualitative nature. In a study on caring for older people in Swedish nursing homes, family members wanted to have a dialog with healthcare staff when they participated in care meetings, to represent the ill person, and not just to participate in practical tasks [28, 29]. The noted changes in attitudes might, therefore, also have been influenced by a stronger wish for families to be involved. Notably, however, the effect size was small, so these differences have minor clinical relevance.

The differences in attitudes between the two samples could also be understood in relation to the organizational or systemic changes to Swedish healthcare between the two data collection periods. For example, examinations and treatments conducted in outpatient clinics or primary care facilities have become more common. The average length of stays after a surgical procedure, such as hip and knee replacements, may be one to two days, which decreases family members’ ability to be involved in practical care. However, nurses must prepare and involve family members for at-home care to support patients’ rehabilitation and recovery. On the other hand, research has shown that families’ involvement in care can be conducted in 15 min per hospitalization [30] or in an outpatient context with short interactions [5]. Furthermore, studies in other countries have found that nurses working in primary healthcare might have more positive attitudes toward family involvement than nurses working in hospitals [31, 32].

The next steps in healthcare are eHealth and the move to more digital healthcare, including communicating with patients and family members using various digital tools, such as mobile applications. For example, a research project has developed a mobile application together with family members caring for dementia patients at home, and healthcare professionals have supported caregivers through this mobile application as a communication tool [33]. This digital development will affect how nurses communicate with patients and family members, implying that nursing students must train in communication with different stakeholders (patients, family members, and nurses in other settings of the care pathway) using different tools. The Covid-19 (coronavirus disease 2019) pandemic has forced healthcare professionals around the world to communicate in new ways, and some of these professionals have had to talk with families by phone, causing feelings of discomfort about providing information about a family member’s deteriorating situation over the phone [34]. Other healthcare professionals have had positive experiences engaging families in video-consulting rounds, enabling these professionals to give patients with cancer a sense of family involvement [35]. Collect such experiences is important to guide future nursing practices.

Nurses’ gender was found to be associated with a significant change in attitudes regarding the examined subscales of viewing families as a resource and conversational partners during the study’s ten years. This result should be considered in light of the study’s relatively few male participants. Three published studies were found to also have included nurses from Sweden in 2015 and 2017. In the 2017 study [21], data from nurses in various countries including Sweden were collected, and the researchers found no association between gender and total scores or any of the subscales. The 2015 study [31] found a significant association between male gender and holding significantly less supportive attitudes toward the total scale and the subscale of viewing families as a resource in nursing care. A study from 2015 exploring the attitudes of Swedish nurses caring for people who needed forensic care in emergency departments [4] found an association between female gender and holding positive attitudes toward family involvement. Two of these studies included nurses caring for patients with cardiovascular diseases and patients in an acute care setting. Whether a specific medical condition or context has a special, gendered mediating effect on nurses’ attitudes remains an open question, and further research should explore this question more deeply.

Nurses’ age was positively associated with positive attitudes across years in the current study, both in relation to the FINC-NA total scale and all subscales. Other studies have also found that older ages are associated with more positive attitudes, and this finding might relate to feeling more competent in the nursing role and having experienced positive outcomes when involving families [36]. Another explanation could be that more older nurses hold higher educational degrees in nursing than younger nurses, and older nurses may have experienced a serious illness among their own families, which would be consistent with earlier findings among Swedish nurses [14], as well as nurses in Iceland [20], Taiwan [18], and Denmark [16].

### Limitations

The study faced some limitations that must be considered. The virtual snowball sampling used for the 2019 sample offered many advantages, including an expanded sample size, reduced costs, flexible response times, and reduced missing items since a question had to be answered before respondents could advance to the next question. This referral approach was more likely to increase response rates compared to other forms of sampling [37]. However, the disadvantages of this approach include a perception of the questionnaire as a spam message unless it is well described, selection bias related to the internet population, an impersonal nature, and respondents’ inability to answer questions if the instructions are unclear [38, 39].

Moreover, we did not include information about participants’ care organizations or how many years they had worked since graduating, both of which have been found to be significantly associated with attitudes toward involving families in care in Swedish contexts [14]. Relatedly, the 2019 study collected different background characteristics than the 2009 study. Only *age*, *sex*, and *own experience of a seriously ill family member* were shared between the two studies. Therefore, the two samples may have differed in other important aspects. To address the problem of the 2019 sample’s significantly older sample and greater personal experiences of seriously ill family members, these variables—together with *gender*—were controlled in our regression analysis. However, other variables could have been important to better compare the two samples. Finally, the authors of the FINC-NA have recommended that the scale’s total score should be the sum of item-response scores within each subscale. Because the subscales include different numbers of items, the scale scores were not comparable. To resolve this problem in the present study, we used linear transformation by dividing the sum of item responses in each subscale by the number of items, resulting in a scale score of 1–5. Future studies can use this transformation approach if they must also compare the subscales. If our approach is adopted, we strongly recommend also reporting the original sum scores to make the results comparable with other studies that have used the FINC-NA.

## Conclusion

Swedish nurses’ overall attitudes toward involving families in care did not change over ten years, maintaining the same levels despite changes in nursing, healthcare-system, and society.

## Data Availability

The datasets generated and/or analysed during the current study are not publicly available due to limitations of ethical approval involving the patient data and anonymity but are available from the corresponding author on reasonable request.

## References

[1] Østergaard B, Mahrer-Imhof R, Wagner L, Barington T, Videbæk L, Lauridsen J (2018). Effect of family nursing therapeutic conversations on health-related quality of life, self-care and depression among outpatients with heart failure: A randomized multi-centre trial. Patient Educ Couns.

[2] Petursdottir AB, Sigurdardottir V, Rayens MK, Svavarsdottir EK (2020). The Impact of Receiving a Family-Oriented Therapeutic Conversation Intervention Before and During Bereavement Among Family Cancer Caregivers: A Nonrandomized Trial. J Hosp Palliat Nurs JHPN Off J Hosp Palliat Nurses Assoc.

[3] Petriwskyj A, Gibson A, Parker D, Banks S, Andrews S, Robinson A (2014). Family involvement in decision making for people with dementia in residential aged care: a systematic review of quantitative literature. Int J Evid Based Healthc.

[4] Linnarsson JR, Benzein E, Årestedt K (2015). Nurses’ views of forensic care in emergency departments and their attitudes, and involvement of family members. J Clin Nurs.

[5] Voltelen B, Konradsen H, Østergaard B (2016). Family Nursing Therapeutic Conversations in Heart Failure Outpatient Clinics in Denmark: Nurses’ Experiences. J Fam Nurs.

[6] Murcia SEA, Lopez L. The experience of nurses in care for culturally diverse families: A qualitative meta-synthesis. Rev Lat Am Enfermagem. 2016;24:e2718. 10.1590/1518-8345.1052.2718 PMC4964299 27384469

[7] Benzein E, Johansson B, Saveman B-I (2004). Families in home care–a resource or a burden?. District nurses’ beliefs J Clin Nurs.

[8] Mackie BR, Marshall A, Mitchell M (2018). Acute care nurses’ views on family participation and collaboration in fundamental care. J Clin Nurs.

[9] Hoplock L, Lobchuk M, Dryburgh L, Shead N, Ahmed R (2019). Canadian Hospital and Home Visiting Nurses’ Attitudes Toward Families in Transitional Care: A Descriptive Comparative Study. J Fam Nurs.

[10] Kapborg I (1998). Nursing education in Sweden: development from vocational training to higher level education. J Adv Nurs.

[11] Lindberg M, Carlsson M, Engström M, Kristofferzon M-L, Skytt B (2020). Nursing student’s expectations for their future profession and motivating factors - A longitudinal descriptive study from Sweden. Nurse Educ Today.

[12] Saveman B-I (2010). Family nursing research for practice: the Swedish perspective. J Fam Nurs.

[13] Benzein E, Johansson P, Arestedt KF, Berg A, Saveman B-I (2008). Families’ Importance in Nursing Care: Nurses’ Attitudes–an instrument development. J Fam Nurs.

[14] Benzein E, Johansson P, Arestedt KF, Saveman B-I (2008). Nurses’ attitudes about the importance of families in nursing care: a survey of Swedish nurses. J Fam Nurs.

[15] Alfaro Diaz C, Esandi Larramendi N, Gutierrez-Aleman T, Canga-Armayor A (2019). Systematic review of measurement properties of instruments assessing nurses’ attitudes towards the importance of involving families in their clinical practice. J Adv Nurs.

[16] Østergaard B, Clausen AM, Agerskov H, Brødsgaard A, Dieperink KB, Funderskov KF (2020). Nurses’ attitudes regarding the importance of families in nursing care: A cross-sectional study. J Clin Nurs.

[17] Luttik M, Goossens E, Ågren S, Jaarsma T, Mårtensson J, Thompson DR (2017). Attitudes of nurses towards family involvement in the care for patients with cardiovascular diseases. Eur J Cardiovasc Nurs J Work Group Cardiovasc Nurs Eur Soc Cardiol.

[18] Hsiao C-Y, Tsai Y-F (2015). Factors Associated With the Perception of Family Nursing Practice Among Mental Health Nurses in Taiwan. J Fam Nurs.

[19] Angelo M, Cruz AC, Mekitarian FFP, Santos CC, da SD, Martinho MJCM, Martins MMFP, da S (2014). Nurses’ attitudes regarding the importance of families in pediatric nursing care. Rev Esc Enferm U P.

[20] Blöndal K, Zoëga S, Hafsteinsdottir JE, Olafsdottir OA, Thorvardardottir AB, Hafsteinsdottir SA (2014). Attitudes of Registered and Licensed Practical Nurses About the Importance of Families in Surgical Hospital Units: Findings From the Landspitali University Hospital Family Nursing Implementation Project. J Fam Nurs.

[21] Statista. Number of hospital beds in Sweden from 2000 to 2018 [Internet]. 2021 [cited 2021 Feb 17]. Available from: https://www.statista.com/statistics/557358/hospital-beds-in-sweden/

[22] OECD. Health at a glance: Europe [Internet]. 2018. Available from: https://www.oecd.org/health/health-at-a-glance-europe-23056088.htm

[23] Schön P, Lagergren M, Kåreholt I (2016). Rapid decrease in length of stay in institutional care for older people in Sweden between 2006 and 2012: results from a population-based study. Health Soc Care Community.

[24] Park M, Giap T-T-T (2020). Patient and family engagement as a potential approach for improving patient safety: A systematic review. J Adv Nurs.

[25] Saveman B-I, Benzein EG, Engström ÅH, Årestedt K (2011). Refinement and psychometric reevaluation of the instrument: Families’ Importance In Nursing Care–Nurses’ Attitudes. J Fam Nurs.

[26] Cohen J (1988). Statistical power analysis for the behavioral sciencesC.

[27] Whitaker C, Stevelink S, Fear N (2017). The Use of Facebook in Recruiting Participants for Health Research Purposes: A Systematic Review. J Med Internet Res.

[28] Ekström K, Spelmans S, Ahlström G, Nilsen P, Alftberg Å, Wallerstedt B (2019). Next of kin’s perceptions of the meaning of participation in the care of older persons in nursing homes: a phenomenographic study. Scand J Caring Sci.

[29] Wallerstedt B, Behm L, Alftberg Å, Sandgren A, Benzein E, Nilsen P (2018). Striking a Balance: A Qualitative Study of Next of Kin Participation in the Care of Older Persons in Nursing Homes in Sweden. Healthc Basel Switz.

[30] dos Santos Ribeiro MCL, Moules NJ, Silva L, Bousso RS (2013). The 15-minute family interview: a family health strategy tool. Rev Esc Enferm U P.

[31] Gusdal AK, Josefsson K, Thors Adolfsson E, Martin L (2017). Nurses’ attitudes toward family importance in heart failure care. Eur J Cardiovasc Nurs J Work Group Cardiovasc Nurs Eur Soc Cardiol.

[32] Hagedoorn EI, Paans W, Jaarsma T, Keers JC, van der Schans CP, Luttik MLA (2020). The importance of families in nursing care: attitudes of nurses in the Netherlands. Scand J Caring Sci.

[33] Kabir ZN, Leung AYM, Grundberg Å, Boström A-M, Lämås K, Kallström AP (2020). Care of family caregivers of persons with dementia (CaFCa) through a tailor-made mobile app: study protocol of a complex intervention study. BMC Geriatr.

[34] Sindhu KK (2021). The Phone: Communication in the Age of COVID-19. Patient Educ Couns.

[35] Petersson NB, Jørgensen AL, Danbjørg DB, Dieperink KB (2020). Video-consulted rounds with caregivers: The experience of patients with cancer. Eur J Oncol Nurs Off J Eur Oncol Nurs Soc.

[36] Duhamel F, Dupuis F, Turcotte A, Martinez A-M, Goudreau J (2015). Integrating the Illness Beliefs Model in clinical practice: a Family Systems Nursing knowledge utilization model. J Fam Nurs.

[37] Mirabeau L, Mignerat M, Grangé C. The utility of using social media networks for data collection in survey research [Internet]. 2013. Available from: https://aisel.aisnet.org/cgi/viewcontent.cgi?article=1313&context=icis2013

[38] Alshaikh F, Ramzan F, Rawaf S, Majeed A (2014). Social network sites as a mode to collect health data: a systematic review. J Med Internet Res.

[39] F Baltar, I Brunet. Social research 2.0: virtual snowball sampling method using Facebook. 2012;22(1). Available from: https://www.emerald.com/insight/content/doi/10.1108/10662241211199960/full/html

